# The Intracellular Threonine of Amyloid Precursor Protein That Is Essential for Docking of Pin1 Is Dispensable for Developmental Function

**DOI:** 10.1371/journal.pone.0018006

**Published:** 2011-03-22

**Authors:** Alessia P. M. Barbagallo, Zilai Wang, Hui Zheng, Luciano D'Adamio

**Affiliations:** 1 Department of Microbiology and Immunology, Einstein College of Medicine, Bronx, New York, United States of America; 2 Huffington Center on Aging, Baylor College of Medicine, Houston, Texas, United States of America; 3 Institute of Neurobiology and Molecular Medicine, CNR, Rome, Italy; Nathan Kline Institute and New York University School of Medicine, United States of America

## Abstract

**Background:**

Processing of Aβ-precursor protein (APP) plays an important role in Alzheimer's Disease (AD) pathogenesis. Thr residue at amino acid 668 of the APP intracellular domain (AID) is highly conserved. When phosphorylated, this residue generates a binding site for Pin1. The interaction of APP with Pin1 has been involved in AD pathogenesis.

**Methodology/Principal Findings:**

To dissect the functions of this sequence *in vivo*, we created an *APP* knock-in allele, in which Thr^668^ is replaced by an Ala (T^668^A). Doubly deficient *APP*/*APP-like protein 2* (*APLP2*) mice present postnatal lethality and neuromuscular synapse defects. Previous work has shown that the APP intracellular domain is necessary for preventing early lethality and neuromuscular junctions (NMJ) defects. Crossing the T^668^A allele into the *APLP2* knockout background showed that mutation of Thr^668^ does not cause a defective phenotype. Notably, the T^668^A mutant APP is able to bind Mint1.

**Conclusions/Significance:**

Our results argue against an important role of the Thr^668^ residue in the essential function of APP in developmental regulation. Furthermore, they indicate that phosphorylation at this residue is not functionally involved in those APP-mediated functions that prevent (NMJ) defects and early lethality in *APLP2* null mice.

## Introduction

Amyloid-β-precursor protein (APP) plays an important role in Alzheimer's Disease (AD) pathogenesis [1], [2], [3], [4], [5], [6], [7], [8], [9], [10]. The prevalent Amyloid cascade hypothesis of AD pathogenesis posits that dementia is caused by Aβ aggregates. The repeated failure of therapeutic approaches based on this dogma in humans suggests that alterations of normal APP functions may contribute to AD pathogenesis. Thus, understanding the role of APP *in vivo* is much needed to reveal fundamental insights into AD pathogenesis and develop potential therapeutic intervention.

*APP* null mice have given scant information about the functions of APP and these mice exhibit seizures, impaired grip strength, locomotor activity, exploratory activity, cognition and LTP [11], [12], [13], [14], [15], [16], [17], [18], [19], [20], [21], [22], [23], [24]. *APP Like Protein 1* and *2* (*APLP1* and *ALPL2*), which belong to the *APP* gene family, are structurally [25], [26] and functionally similar to APP. The evidence that *APLP1^−/−^, APLP2^−/−^, APP^−/−^* and *APLP1^−/−^APP^−/−^* mice are viable, whereas combined *APP^−/−^APLP2^−/−^* or *APLP1^−/−^APLP2^−/−^* double KO [27], [28] die shortly after birth show that functional redundancy compensates for the loss of essential gene functions in APP knock out mice. Analysis of *APP*/*APLP2* double knockout (dKO) mice uncovered an essential role for the APP and APLP2 in the patterning of neuromuscular junction (NMJ) [29], [30], [31].

Recent evidence shows that the synaptogenic function of APP requires the highly conserved intracellular domain [32], and in particular Tyr^682^ [33]. This residue is part of the YENPTY sequence (amino acids 682–687, following the numbering of 695 amino acids long brain APP isoform), which is a docking site for numerous cytosolic proteins [34], [35], [36], [37], [38], [39], [40], [41], [42], [43]. Some proteins, such as Grb2 [44], [45], Shc [45], [46], Grb7 and Crk [47] interact with APP only when Tyr^682^ is phosphorylated; others, like Fe65, Fe65L1 and Fe65L2 only when this tyrosine is not phosphorylated [48].

Thr^668^ is another conserved residue of the intracellular region of APP. This residue has been intensively studied as phosphorylation of Thr^668^ promotes Pin1 binding [49]. The interaction of Pin1 with APP has been shown to reduce APP processing and Aβ generation, thereby protecting from AD [50]. These data are not easily reconcilable with other evidence showing that mutation of Thr^668^ in vivo does not grossly alter APP processing [51], [52]. Here, we asked whether Thr^668^ and its phosphorylation plays an important physiological role. The NMJ deficits and the early postnatal lethality present in the *APP/APLP2* double knockout animals provide genetic readouts to determine the role of this amino acid *in vivo*. We have created an *APP* knock-in (ki) mouse in which Thr^668^ is replaced by an alanine (we will refer to these mice as *APP^TA^*), thereby abolishing phosphorylation at this position. We report that *APP^TA/TA^/APLP2^−^* ^/*−*^ mice, unlike *APP/APLP2* double KO mice, do not present NMJ deficits and early lethality, demonstrating that phosphorylation of Thr^668^ is dispensable for the essential function of APP in developmental regulation.

## Materials and Methods

### Mice and Ethics Statement

Mice were maintained on a C57BL/6 background for several generations (at least 15). Mice were handled according to the Ethical Guidelines for Treatment of Laboratory Animals of Albert Einstein College of Medicine. The procedures were described and approved by the Institutional Animal Care and Use Committee (IACUC) at the Albert Einstein College of Medicine in animal protocol number 20040707. APP-ki generation and genotyping has been described [51]. Genotyping for the APP and APLP2 KO alleles were performed as described in the Jackson Laboratory WEB site.

### Mouse brain preparations and GST pull-down experiments

Brains were homogenized (w/v =  10 mg tissue/100 ml buffer) in tissue homogenization buffer (20 mM Tris-base pH 7.4, 1 mM EDTA, 1 mM EGTA) supplemented with protease and phosphatase inhibitors (PI and PhI). The post nuclear supernatant (PNS) was prepared by precipitating the nuclei and debris by centrifuging the homogenates at 1000 g for 10 min. GST fusion proteins were produced and purified as described [38]. The binding experiments were performed using ∼6 µg (200 pmol) of GST or GST-Mint1 PTB [47] following the methods described previously [38]. To detect the bound APP we used the 22C11 (Chemicon) antibody in Western blot analysis.

### Immunofluorescence staining

The muscle dissection, preparation, staining, and quantification of the neuromuscular synapses have been previously described [29], [31]. Confocal images were obtained with a Zeiss 510 laser-scanning microscope, and quantification was done using the ImageJ program from NIH. Antibodies: anti-synaptophysin (Dako, 1∶500); Anti-neurofilament (DSHB 1∶500); anti-APP (Epitomics Inc., Y188, 1∶250); anti-Alexa-488/555/647 conjugated secondaries and α-bungarotoxin (Molecular Probe).

### Statistical Analysis

Genotyping analysis of the offspring from APP^ki/−^APLP2^+/−^ male and female intercrosses was performed using χ^2^ analysis. The Student's t test was used for all other analyses (*P<0.05; **P<0.01; ***P<0.001). Data were presented as the average ± SEM.

## Results and Discussion

### Expression of *APP^TA^* on *APLP2* null background does not lead to early postnatal lethality

**Figure 1:**
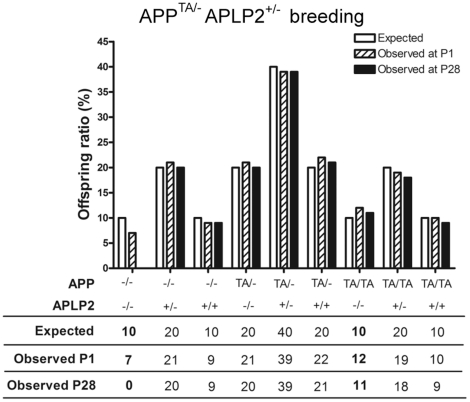
Survival analysis of *APP^TA^* ki mice on *APLP2* null background. Analysis of genotypes of 128 offspring collected at P1 derived from crosses of *APP^TA/−^APLP2^+/−^* male and female mice. All genotypes were recovered at close to a Mendelian ratio (df = 8, p>0.95). Analysis of genotypes of these same offspring at P28 showed that the number of *APP^−/−^APLP2^−/−^* animals observed was much lower than expected (highlighted in bold, df = 8, p<0.001). On the contrary, the number of *APP^TA/TA^APLP2^−/−^*, *APP^TA/−^APLP2^−/−^* was still close to a Mendelian ratio (B, df = 8, p>0.20).

We tested whether *APLP2* KO mice carrying the *APP^TA^* mutation have a lethal phenotype, similar to the *APP/APLP2* dKO mice. This genetic approach is ideal to assess whether the Thr^668^ mediates the essential functions of APP. We inter-crossed double heterozygous mice harboring one allele each of the *APP* and *APLP2* null mutations (*APP^TA/−^APLP2^+/−^*). We then determined the genotypes of the offspring at postnatal day 1 (P1) and day 28 (P28), and compared the number observed against the number expected (Figure 1A and B). Genotyping of P1 pups revealed a close to Mendelian distribution of all genotypes, indicating no embryonic lethality as expected (Chi square analysis: at P1, ∑d^2^/e =  1.775, df = 8, p>0.95). The distribution of *APP^TA/TA^APLP2^−/−^* and *APP^TA/−^APLP2^−/−^* mice did not change between age P1 and P28 (Chi square analysis at P28, ∑d^2^/e =  10.575, df = 8, p>0.20) (Figure 1). These results demonstrate that mutation of Thr^668^ into an Ala does not affect the essential functions of APP that, when compromised, lead to postnatal lethality of the *APP/APLP2* double deficient mice. This is in sharp contrast with what is observed in mice with deletion of the entire APP intracellular domain or a point mutation of Tyr^682^, another conserved residues in the AID, which show early lethality similarly to *APP^−/−^APLP2^−/−^* animals [32], [33].

### Analysis of neuromuscular synapses development in *APP^TA^* knock-in animals

**Figure 2:**
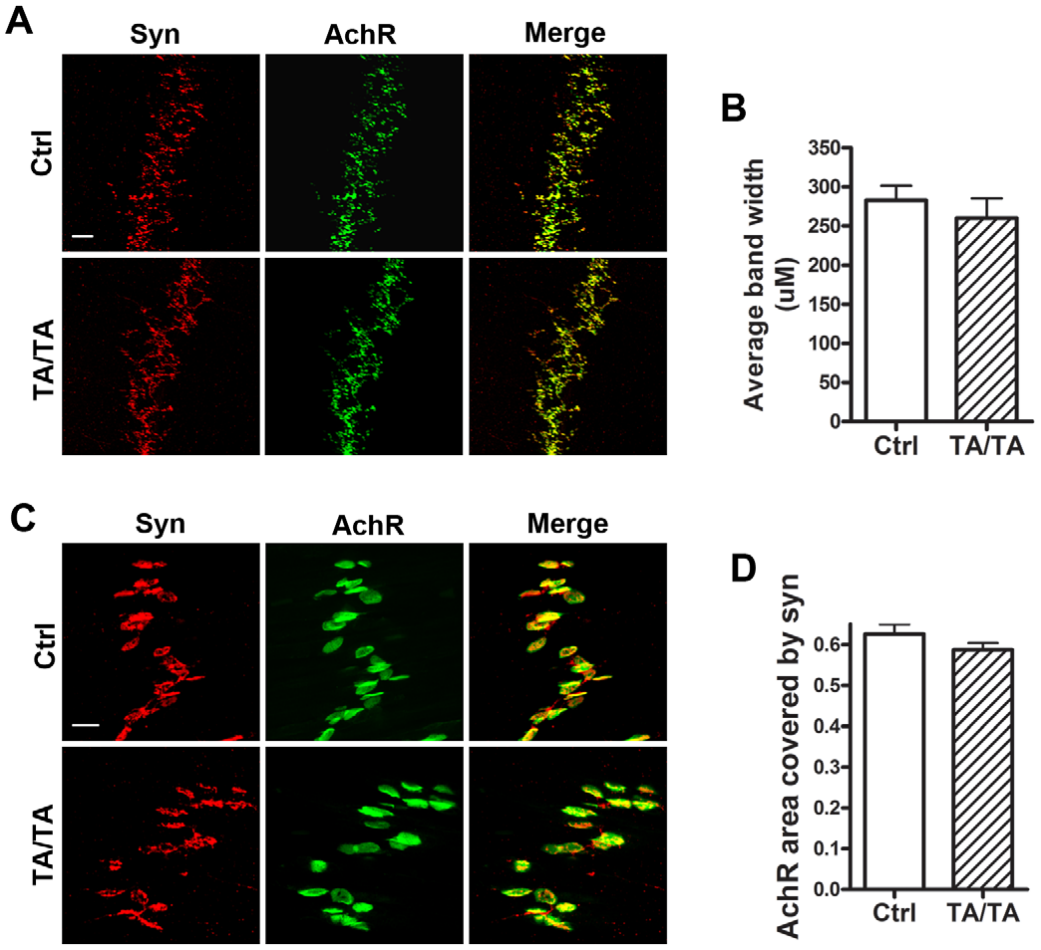
No obvious neuromuscular synapse defects were observed in *APP^TA/TA^/APLP2^−/−^* mice. **A**. Whole-mount staining of littermate *APP^+/+^/APLP2^−/−^*control (Ctrl) and *APP^TA/TA^/APLP2^−/−^* (TA) P0 diaphragm muscles with antibodies against synaptophysin (Syn). Anti-BTX was used to mark the AchRs. **B**. Quantification of the average band width of endplates marked by anti-BTX (band width in control 260.0±25.17 µm vs. TA 282.9±18.63 µm. p>0.05, student t-test. Mean ± SEM of 3 animals/genotype). **C**. Higher magnification images showing endplates closely apposed by Syn and no axonal Syn staining in the TA mutant. **D**. Quantification of the area percentage of AchR endplates covered by Syn (control 0.626±0.024 vs. TA 0.588±0.017. p>0.05, student t-test. Mean±SEM of 20 endplates/genotype). Scale bar: A, 100 µm; C, 20 µm.

*APP^−/−^APLP2^−/−^* animals show dramatic defects in the development of NMJ [29], [31]. This phenotype, just like the postnatal early letality, requires the APP intracellular domain and Tyr^682^ [32], [33]. Analysis of the neuromuscular synapse at P0 stage showed that *APP^TA/TA^APLP2^−/−^* mutants exhibited normal neuromuscular synapses, compared to *APP* ^+/+^ *APLP2^−^* ^/*−*^ littermate controls (Figs. 2A and 2C and quantified in 2B and 2D). These results demonstrate that, in agreement with the survival result, Thr^668^ is not involved in the NMJ analysis of *APP^TA/TA^APLP2^−/−^* mutants revealed indistinguishable staining patterns compared to the littermate *APP^+/+^APLP2^−/−^* controls expressing wild-type APP (Figure 2A and C).

### The TA mutation does not affect the APP/Mint1 interaction

**Figure 3:**
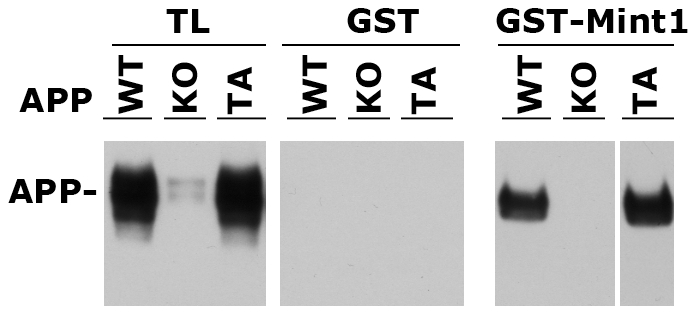
APP^TA^ interacts with Mint1. GST-Mint1 pull-down experiments show that Mint1 interacts with both wild-type APP (WT) and APP^TA^ (TA) mutant. The interaction is specific since GST alone does not interact with APP and GST-Mint1 does not pull down a protein of size similar to APP and cross-reacting with the anti-APP antibody 22C11 from brain lysates of APP KO (KO) mice. The faint doublet recognized by the 22C11 in the total lysates (TL) from APP KO mice probably represents low levels of cross-reactivity of the antibody with APLP1 and/or APLP2.

It has been proposed that presynaptic differentiation induced by APP involves intracellular association with Cask and Mint1 [31], similarly to neurorexin/neuroligin (NX/NL) and SynCAM class of synaptic adhesion proteins [53], [54], [55]. We asked whether mutation of Thr^668^ interfered with the formation of a Mint1/APP complex. To test for this, we produced a recombinant protein *in vitro*, in which the PTB domain of Mint1 was fused to GST for production and purification from bacterial cultures. As a control we produced GST on its own [47]. These recombinant proteins were used for pull down experiments from mouse brain lysates. GST-Mint1 interacts with APP in samples isolated from both WT mice and mice expressing the APP^TA^ mutant form. The interaction is specific since GST does not bring down APP and a molecule reacting with the anti-APP antibody is not isolated by GST-Mint1 when brain lysates from APP KO mice are used (Fig. 3). These results support the view that the Thr^668^ mutation does not abolish presynaptic functions of APP because it does not impair the recruitment of Mint1.

The highly conserved APP intracellular region is required for APP-mediated survival and neuromuscular synapse assembly *in vivo*, and Tyr^682^ is necessary for these functions of APP [32], [33]. We report here that, in contrast, mutant APP mice with a non-phosphorylatable alanyl residue at position 668, have preserved essential APP functions. The non-consequential effects of the mutation on Thr^668^ suggest that the APP/Pin1 interaction does not play a role in signaling pathways that regulate survival and synaptogenesis.

Thr668 is followed by a Pro, which generates a consensus site for phosphorylation, in APP family members and in other species, except for APLP1 and *Drosophila* APP ortologue. Phosphorylation of APP at Thr^668^ impairs Fe65 interaction [47], [56] while promotes Pin1 binding [49] **.** Pin1 is a prolyl isomerase that regulates protein function by accelerating conformational changes. It has been reported that Pin1 is downregulated and/or inhibited by oxidation in Alzheimer's disease neurons [57]. Moreover, Pin1 knockout causes tauopathy and neurodegeneration [58], and increased amyloidogenic APP processing [50]. These findings have lead to propose that phosphorylation of Thr^668^ protects from AD by promoting interaction with Pin1, which in turn has a protective effect against amyloidosis and tauopathy. These conclusions however seem to be contradicted by evidence showing that *APP^TA^* knock-in mice, in which phosphorylation at position 668 is suppressed, show levels of Aβ comparable to wild type mice, suggest that Thr^668^ phosphorylation does not play an obvious role in governing the physiological levels of brain Aβ *in vivo* [51], [52].

The evolutionary pressure that has resulted in conservation of this residue during evolution of APP denotes the importance of Thr^668^ for APP functions. However, the finding that the *APP^TA^* mutation rescues NMJ and lethality of APP/APLP2 deficient mice, argues against an essential function for phosphorylation of this residue. The synaptic promoting property of APP may involve the formation of the APP/Mint1/Cask complex in pre-synaptic termini [31]. Mint1 belongs to a gene family that comprises also Mint3. Both Mint1 and 3 bind APP and have opposite effects on the localization of AID [59]. Here we focused on Mint1 because only Mint1 interacts with CASK. In the trans-synaptic interaction model we have previously proposed for APP function in sysnaptogenesis, APP-Mint1-CASK is likely the central complex mediating APP effect [31]. As discussed, Mint1-CASK complex has also been implicated in Neurexin-Neuroligin mediated signaling in presynaptic organization [53], [54], [55]. The finding that Mint1 binds both WT and APP^TA^ mutant but not APP^YG^ [51] backs this hypothesis.

Growing evidence supports a role for alteration of synaptic function in AD. Our previous finding that the intracellular region and Tyr^682^ of APP plays a role in synaptogenesis makes it a legitimate possibility that the APP intracellular domain may contribute to AD pathogenesis [32], [51]. If phosphorylation of Thr^668^ has a protective role in AD pathogenesis, the finding that *APP^TA^*/*ALPL2^−/−^* mice do not present NMJ development defects represents a notable exception to this hypothesis. However, it is still possible that Thr^668^ and its phosphorylation may functionally regulate synapses in the Central nervous system, especially those involved in memory formation in the hippocampus. To answer these questions, it will be important to unveil the biological mechanisms that regulate phosphorylation of APP on Thr^668^ and the signaling pathways that are controlled by this functional domain of APP. In addition, Thr^668^ and its phosphorylation may regulate signaling pathway that are distinct by those that when compromised double mutant mice, lead to early lethality and NMJ dysfunctions. It is also conceivable that those roles of Thr^668^ may play a pathogenic role in AD.
